# Supplementary material for: Causal Relationship between Aging and Anorexia Nervosa: A White-Matter-Microstructure-Mediated Mendelian Randomization Analysis
Source: Biomedicines. 2024 Aug 16;12(8):1874. doi: 10.3390/biomedicines12081874 (PMC11351342; doi:10.3390/biomedicines12081874)
Supplement: Supplementary file 1 [file biomedicines-12-01874-s001.zip › Supplementary Figure/Figure S4/Singe SNP plot of MR analysis for TL to ubm-b-2039.pdf]

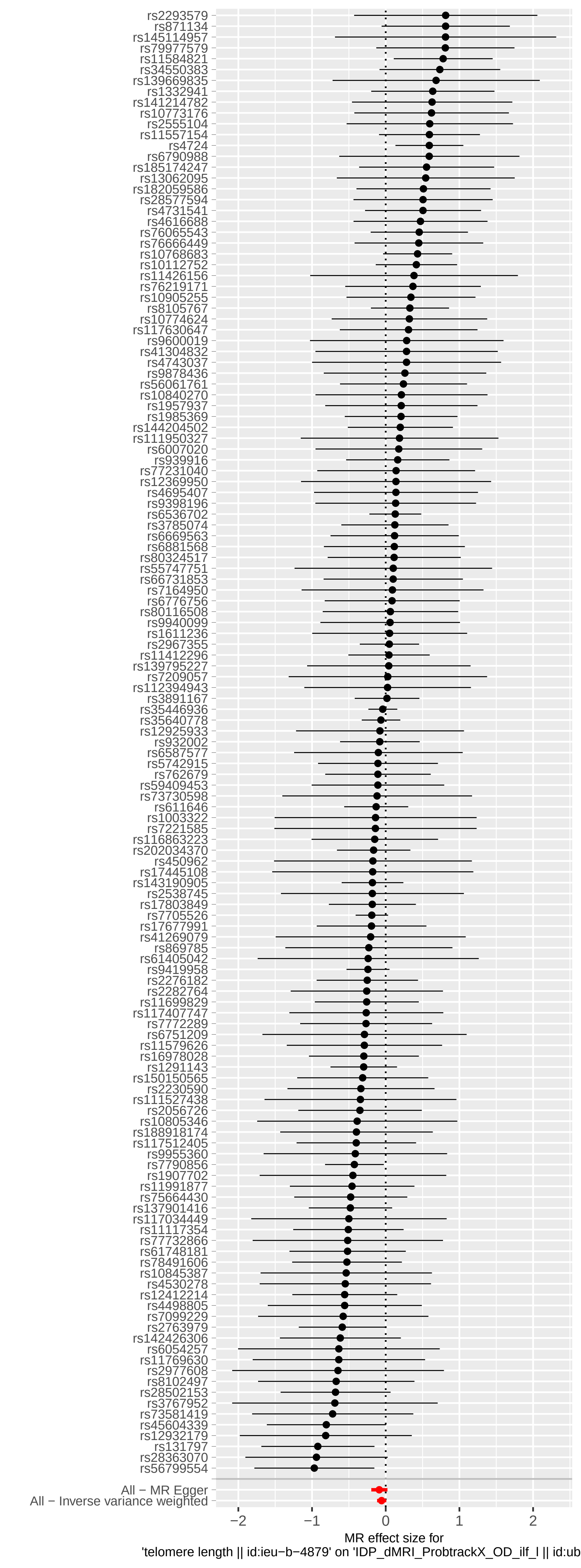
rs2293579
rs871134
rs145114957
rs79977579
rs11584821
rs34550383
rs139669835
rs1332941
rs141214782
rs10773176
rs2555104
rs11557154
rs4724
rs6790988
rs185174247
rs13062095
rs182059586
rs28577594
rs4731541
rs4616688
rs76065543
rs76666449
rs10768683
rs10112752
rs11426156
rs76219171
rs10905255
rs8105767
rs10774624
rs117630647
rs9600019
rs41304832
rs4743037
rs9878436
rs56061761
rs10840270
rs1957937
rs1985369
rs144204502
rs111950327
rs6007020
rs939916
rs77231040
rs12369950
rs4695407
rs9398196
rs6536702
rs3785074
rs6669563
rs6881568
rs80324517
rs55747751
rs66731853
rs7164950
rs6776756
rs80116508
rs9940099
rs1611236
rs2967355
rs11412296
rs139795227
rs7209057
rs112394943
rs3891167
rs35446936
rs35640778
rs12925933
rs932002
rs6587577
rs5742915
rs762679
rs59409453
rs73730598
rs611646
rs1003322
rs7221585
rs116863223
rs202034370
rs450962
rs17445108
rs143190905
rs2538745
rs17803849
rs7705526
rs17677991
rs41269079
rs869785
rs61405042
rs9419958
rs2276182
rs2282764
rs11699829
rs117407747
rs7772289
rs6751209
rs11579626
rs16978028
rs1291143
rs150150565
rs2230590
rs111527438
rs2056726
rs10805346
rs188918174
rs117512405
rs9955360
rs7790856
rs1907702
rs11991877
rs75664430
rs137901416
rs117034449
rs11117354
rs77732866
rs61748181
rs78491606
rs10845387
rs4530278
rs12412214
rs4498805
rs7099229
rs2763979
rs142426306
rs6054257
rs11769630
rs2977608
rs8102497
rs28502153
rs3767952
rs73581419
rs45604339
rs12932179
rs131797
rs28363070
rs56799554
All − MR Egger
All − Inverse variance weighted
−2
−1
0
1
2
MR effect size for
'telomere length || id:ieu−b−4879' on 'IDP_dMRI_ProbtrackX_OD_ilf_l || id:ub
